# Tracheotomy Successfully Performed for the Removal of a Grain of Corn, Which Had Remained Seven Weeks in the Trachea and Had Commenced to Germinate

**Published:** 1856-08

**Authors:** Henry H. Smith

**Affiliations:** Professor of Surgery in University of Pennsylvania


*Tracheotomy successfully performed for the removal of a grain of corn, which had remained seven weeks in the trachea and had commenced to germinate.* By HENRY H. SMITH, M. D., Professor of Surgery in University of Pennsylvania. Reported by EDWARD SHIPPEN, A. M., Philadelphia.

As the usefulness of statistics in determining the result of surgical operations depends upon all the cases being recorded, this is reported in order to afford another instance of the success of tracheotomy performed for the removal of foreign substances from the air passages. Showing that the operation on an uninflamed trachea is most frequently followed by favorable results, though in croup its success is only in the proportion of about one in five.

The patient G., from Delaware, a boy about four years old, of robust health, whilst playing with some corn, threw a grain of it into his mouth, and soon after became black in the face, and evinced other signs of strangulation. In a few seconds respiration returned and the spasm passed off, leaving him exhausted, with impaired voice and subject to frequent spells of spasmodic cough of great violence, and repeated attacks of strangulation. As the corn was not dislodged by the emetics given by the attending physician, and as the cough increased, the mother was advised to bring the child to Philadelphia for surgical treatment.

Upon Dr. Smith being called in, the patient was found indisposed to speak and with a disposition to cough on the least exertion, coughing spasmodically, not unlike the cough in pertussis, with a loose râle that could be heard by any one near him, and pointing to a spot near his right nipple as the seat of his pain. On ausculting the chest, loose mucous and loud sibilant râles concealed every other sign of the disorder. These râles were heard on both sides, but were more distinct on the right. A careful examination failed to obtain any positive proof of the whereabouts of the corn, and after a few days watching, noting the character of the spasms of coughing, the lividity of the countenance and the subsequent exhaustion, Dr. Smith gave his consent to the performance of tracheotomy, believing the rational signs, with the history of the case, to be sufficiently positive evidence that the grain of corn was yet in the trachea.

Accordingly, on the 5th of April the operation was performed in the presence of numerous members of the University class, the child being in a state of anæsthesia. After having checked the bleeding from the turgid veins, consequent upon the first incisions by the application of two ligatures, the trachea was opened from the first to the fourth ring, and the grain of corn seized as it rose to the orifice during the spasm consequent upon the puncture of the trachea. About two drachms of pure pus were ejected from the trachea at the same moment. The spasm having passed off, a narrow strip of pewter was passed around the neck from behind forwards, and the ends being brought to the edges of the wound were bent down so as to serve as blunt hooks and keep the muscles and integuments of each side of the wound widely apart, thus securing a patulous wound and preventing leakage into the trachea. The child being placed in bed soon rallied from the anæsthetic state. A piece of moist gauze was placed over the wound and moistened from time to time by members of the class, who very assiduously watched the patient for 48 hours. In 24 hours the hooks were removed, and the next day the edges of the wound were approximated and the child playing about the room.

Ssix days after the operation he left the city, the wound having granulated very kindly and being nearly closed. On examining the grain of corn immediately after its removal from the trachea, it was found to be much swollen and soft, with a distinct germ or root sprouting from its end.

